# Inhibitory control of hippocampal inhibitory neurons

**DOI:** 10.3389/fnins.2012.00165

**Published:** 2012-11-14

**Authors:** Simon Chamberland, Lisa Topolnik

**Affiliations:** Axis of Cellular and Molecular Neuroscience, IUSMQ, Department of Biochemistry, Microbiology and Bio-informatics, Université LavalQuébec, QC, Canada

**Keywords:** hippocampus, inhibition, interneuron-specific interneuron, GABA, synapse

## Abstract

Information processing within neuronal networks is determined by a dynamic partnership between principal neurons and local circuit inhibitory interneurons. The population of GABAergic interneurons is extremely heterogeneous and comprises, in many brain regions, cells with divergent morphological and physiological properties, distinct molecular expression profiles, and highly specialized functions. GABAergic interneurons have been studied extensively during the past two decades, especially in the hippocampus, which is a relatively simple cortical structure. Different types of hippocampal inhibitory interneurons control spike initiation [e.g., axo-axonic and basket cells (BCs)] and synaptic integration (e.g., bistratified and oriens–lacunosum moleculare interneurons) within pyramidal neurons and synchronize local network activity, providing a means for functional segregation of neuronal ensembles and proper routing of hippocampal information. Thus, it is thought that, at least in the hippocampus, GABAergic inhibitory interneurons represent critical regulating elements at all stages of information processing, from synaptic integration and spike generation to large-scale network activity. However, this raises an important question: if inhibitory interneurons are fundamental for network computations, what are the mechanisms that control the activity of the interneurons themselves? Given the essential role of synaptic inhibition in the regulation of neuronal activity, it would be logical to expect that specific inhibitory mechanisms have evolved to control the operation of interneurons. Here, we review the mechanisms of synaptic inhibition of interneurons and discuss their role in the operation of hippocampal inhibitory circuits.

## Introduction

The cerebral cortex is populated by a large diversity of **GABAergic inhibitory neurons**. These cells represent only 10–20% of the total neuronal population; however, they are able to efficiently control the information flow within cortical circuits (DeFelipe, 1993; Somogyi et al., 1998; Markram et al., 2004). A particularly vast heterogeneity of GABAergic cells has been reported in the CA1 hippocampal region (Klausberger and Somogyi, 2008). The division of labor between different types of CA1 interneurons in sculpting the hippocampal output activity is an area of intensive investigation. Research in this field has been stimulated mainly by the discovery of the tremendous capacity of individual interneuron types to control different domains of glutamatergic principal neurons at precise moments during hippocampal activity that are associated with various brain states (Sik et al., 1995; Ylinen et al., 1995; Miles et al., 1996; Csicsvari et al., 1999; Klausberger et al., 2003, 2004, 2005; Hajos et al., 2004; Pouille and Scanziani, 2004; Gloveli et al., 2005; Tukker et al., 2007; Fuentealba et al., 2008; Klausberger and Somogyi, 2008). Accordingly, to date, most research has focused on the role of GABAergic interneurons in the coordination of the activity of principal cells. However, glutamatergic pyramidal neurons are not the only postsynaptic target of interneurons. Early anatomical and electrophysiological studies provided evidence that GABAergic cells in the hippocampal formation innervate each other (Misgeld and Frotscher, 1986; Lacaille et al., 1987; Kunkel et al., 1988), suggesting that interneurons themselves are controlled via specific inhibitory mechanisms. This was to be expected, as the proper routing of hippocampal information and functional segregation of neuronal ensembles (Buzsaki and Chrobak, 1995) require strong coordination of the inhibition. Therefore, the inhibitory control of inhibitory interneurons should be of primary importance for circuit operation and information processing.

According to previous reports, the inhibition received by interneurons may arise from four principal sources. First, different types of interneurons can be connected by GABAergic synapses (Lacaille and Schwartzkroin, 1988; Cobb et al., 1997; Vida et al., 1998). Second, it was demonstrated that a subgroup of interneurons, the so-called interneuron-specific (IS) interneurons, specializes in innervating exclusively other GABAergic cells (Acsady et al., 1996; Gulyas et al., 1996). Third, long-range GABAergic projections (e.g., originating from the septum or the entorhinal cortex) are involved in the inhibitory control of hippocampal interneurons (Freund and Antal, 1988; Melzer et al., 2012). Finally, some types of interneurons, such as basket cells (BCs), bistratified cells, and neurogliaform cells are also self-connected via functional autapses (Cobb et al., 1997; Pawelzik et al., 2003; Karayannis et al., 2010). Compared with excitatory inputs onto interneurons, the functional organization of their inhibitory inputs has received far less attention. Here, we consider the mechanisms of synaptic inhibition of hippocampal interneurons and discuss the manner via which their complex interactions may take part in the local and regional coordination of neuronal activity. To begin assembling a connectome of hippocampal inhibitory circuits, we first explore the functional and structural evidence of connectivity between various types of interneurons. We also review the limited data available on the major differences in the organization of inhibitory synapses targeting principal cells and interneurons. Finally, we consider the important consequences of interneuron inhibition in the working brain and discuss new venues that should guide future work. It is not our intention to discuss the widespread presence and the important role of electrical coupling between interneurons as this topic has been explored in several fine reviews (Cruikshank et al., 2005; Hestrin and Galarreta, 2005; Söhl et al., 2005; Fukuda, 2007).

## Local connections between interneurons

In different brain areas, interneurons belonging to the same class are often connected by GABAergic synapses. For example, combined electrophysiological and anatomical studies in the neocortex have shown that BCs expressing parvalbumin (PV) form a high number of synapses (7–20) onto each other (Tamas et al., 1998; Galarreta and Hestrin, 2002). Further examples of GABAergic connections between interneurons of the same type have been reported in the cerebellum, thalamic reticular nucleus, and retina (Hausser and Clark, 1997; Sanchez-Vives et al., 1997; Wei et al., 2011). Therefore, it appears that connectivity within the same class of interneurons is a basic property of inhibitory microcircuit organization. Nonetheless, several studies have demonstrated the existence of inhibitory connections between distinct types of interneurons. In the visual cortex, BCs can form a few synapses onto double-bouquet and dendrite-targeting GABAergic cells (Tamas et al., 1998). In addition, in the neocortex, fast-spiking (likely PV-expressing) and somatostatin (SOM)-expressing interneurons are interconnected (Gibson et al., 1999; Hu et al., 2011). In the hippocampus, morphological analysis revealed that pyramidal neurons represent the major postsynaptic target of most interneurons, whereas a small fraction of synaptic contacts (5–15%) from interneurons is made onto other GABAergic cells (Sik et al., 1995; Cobb et al., 1997). Below, we analyze the experimental evidence of the existence of connections within morphologically defined classes of interneurons, as well as between different classes. As quantitative neuroanatomical and electrophysiological data from identified interneurons are mostly available for the CA1 hippocampal region, we focus our discussion on this brain area.

**Figure 1:**
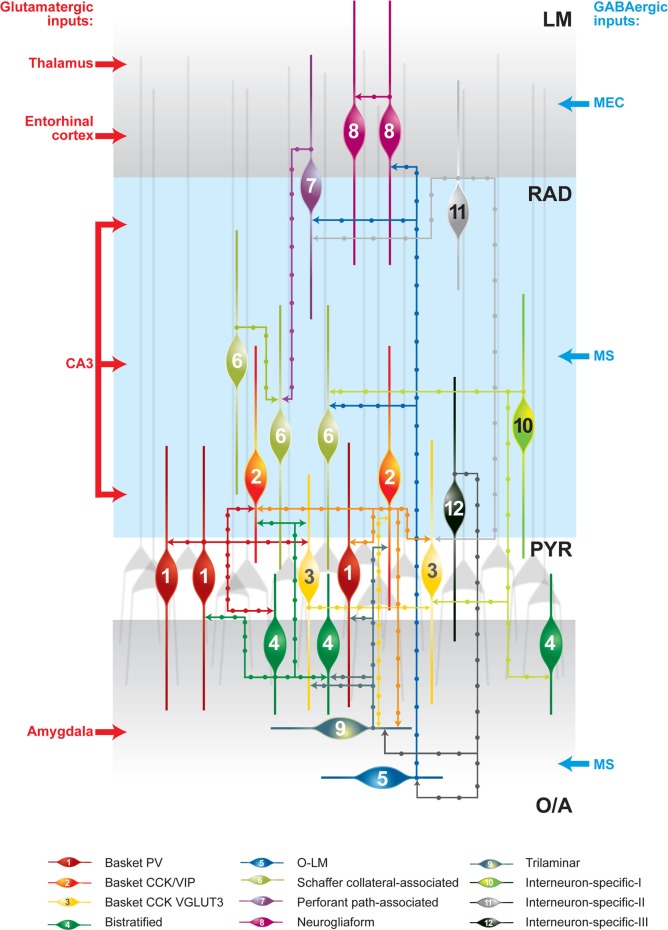
**Schematic representation of synaptically connected GABAergic inhibitory circuits in the CA1 hippocampal area.** The main glutamatergic inputs are indicated on the left. The main long-range GABAergic projections contacting CA1 interneurons are shown on the right. Some connections are indicated on the basis of data from one recording and further analysis may be required. CCK, cholecystokinin; LM, stratum lacunosum moleculare; O/A, stratum oriens/alveus; O–LM, oriens–lacunosum moleculare cell; PV, parvalbumin; PYR, stratum pyramidale; RAD, stratum radiatum; VGLUT3, vesicular glutamate transporter 3; VIP, vasoactive intestinal peptide.

The first direct evidence of synaptic connections between interneurons came from **paired-recording** experiments combined with biocytin labeling and anatomical reconstruction of recorded neurons. In these early experiments, accidental penetration of a postsynaptic interneuron, instead of a principal cell, revealed that BCs can form functional GABAergic synapses onto each other (Figure 1) (Cobb et al., 1997). BCs, with their basket-like axonal arborization surrounding the pyramidal cell soma, provide one of the major sources of perisomatic inhibition to pyramidal neurons in the cortex (Ramon y Cajal, 1893). In the CA1 hippocampus, BCs are further subdivided into three distinct subclasses, based on their neurochemical profile. They may express either PV or cholecystokinin (CCK). In addition, BCs that are positive for CCK may coexpress either the vesicular glutamate transporter 3 or the vasoactive intestinal peptide (VIP) (Somogyi and Klausberger, 2005). The axon of the three types of BCs arborizes almost exclusively within the stratum pyramidale (PYR), where it may contact at least 1500 pyramidal cells, but may also form fewer synapses (up to 12) onto interneurons (Buhl et al., 1994; Sik et al., 1995; Cobb et al., 1997). As it can be predicted from the pattern of axon arborization, GABAergic cells located in the vicinity of the PYR may be contacted by BCs. Indeed, a combination of paired recordings and *post-hoc* anatomical reconstruction in acute hippocampal slices showed that, in addition to being connected with each other, BCs form synapses onto trilaminar cells (Ali et al., 1999). Furthermore, PV-positive BCs form synapses onto CCK-positive BCs and *vice versa*, suggesting that the different BC microcircuits are cross-linked (Figure 1) (Karson et al., 2009).

Another example is found in Schaffer collateral-associated cells (SC-ACs), which, in addition to principal cells, target interneurons (Vida et al., 1998; Pawelzik et al., 2002; Ali, 2007, 2011). Interestingly, although the extensive axonal arborization of SC-ACs within the stratum radiatum (RAD) predicts their connectivity with many distinct classes of RAD interneurons, this is not the case, as they contact mainly each other (Figure 1) (Pawelzik et al., 2002; Ali, 2007). Furthermore, oriens–lacunosum moleculare cells (O–LMs), which represent a major component of the CA1 feedback inhibitory circuit and provide inhibition to the distal dendrites of pyramidal neurons (Lacaille and Schwartzkroin, 1988), can form synapses onto GABA-positive material within the LM (Katona et al., 1999). SOM-positive presynaptic terminals in the LM can contact PV-, CCK-, calretinin (CR)-, and VIP-expressing dendrites (Katona et al., 1999), suggesting that O–LMs may innervate different types of interneurons. Electrophysiological recordings combined with *post-hoc* anatomical identification have shown that O–LMs target neurogliaform cells (NGFCs), BCs, SC-ACs, and perforant path-ACs in the LM (Figure 1) (Elfant et al., 2008). In turn, NGFCs form synapses onto pyramidal neurons and interneurons (Vida et al., 1998; Olah et al., 2009). The identity of the GABAergic cells that may be targeted by NGFCs remains to be determined. Importantly, these cells are tightly interconnected with each other via chemical and electrical synapses (Figure 1) (Price et al., 2005).

Together, these data indicate that, as in the neocortex, hippocampal interneurons of the same, as well as different, classes are likely to form reciprocally connected circuits. As a rule, different subtypes of interneurons targeting the soma and proximal dendrites of pyramidal neurons (e.g., BCs and trilaminar and bistratified cells) form a small fraction of synapses with each other. Similarly, interneurons targeting the distal dendrites of principal neurons (e.g., O–LMs and NGFCs) tend to also control other interneurons that are responsible for distal dendritic inhibition. Finally, the populations of soma- and distal-dendrite-targeting interneurons can be also interconnected. For example, O–LMs make monosynaptic connection with LM BCs (Elfant et al., 2008). Moreover, recent experiments using a combination of optogenetic and pharmacogenetic approaches revealed that a population of SOM-positive interneurons, including O–LMs, can be inhibited by selective activation of PV-expressing cells in slices obtained from PV-Cre mice (Lovett-Barron et al., 2012).

## Interneurons specialized to control other interneurons

In addition to being connected with each other, GABAergic cells in the hippocampus may receive synapses from interneurons specialized in the executive control of inhibitory circuits, the so-called **interneuron-specific (IS) interneurons**. First, a combination of immunohistochemistry and anatomical analysis identified three distinct subtypes of IS interneurons in the rat hippocampus (Acsady et al., 1996; Gulyas et al., 1996). Furthermore, the existence of IS cells in the human hippocampus has been confirmed (Urban et al., 2002). Intriguingly, the hippocampus and the superficial cortical layers (layers 1–3) may be the only cortical regions that possess such a highly specialized population of GABAergic cells (Meskenaite, 1997; Gonchar and Burkhalter, 1999; Melchitzky and Lewis, 2008; Caputi et al., 2009).

### Is interneurons type I

Hippocampal IS interneurons type I (IS-Is) have a soma located in the stratum oriens/alveus (O/A), PYR, or RAD and express CR. In addition to innervating other interneurons exclusively, these cells show further preferences, as they avoid PV-expressing BCs and axo-axonic cells and contact calbindin (CB)- and CR-positive interneurons (Figure 1) (Acsady et al., 1996; Gulyas et al., 1996). The prominent feature of IS-Is is the characteristic organization of their dendrites. Morphological analysis revealed that dendrites of different cells come in close apposition with each other to form dendrodendritic junctions. Therefore, in addition to the numerous GABAergic synapses established by these cells onto CR- and CB-positive dendrites, IS-Is are likely to be connected by electrical synapses. As such, clusters of ~15 cells were estimated to be connected by dendrodendritic junctions, suggesting a highly coordinated activity within this population of interneurons.

### Is interneurons type II

IS interneurons type II (IS-IIs) have been found at the border between the RAD and the LM. These interneurons express VIP, but lack CR. They have a vertically oriented cell body, dendrites restricted to the LM, and an axon that arborizes in the RAD. It has been shown that the VIPergic projections in the RAD form synapses onto CB- or VIP-positive dendrites, with a preference for CCK/VIP-coexpressing BCs (Figure 1) (Acsady et al., 1996).

### Is interneurons type III

**Figure 2:**
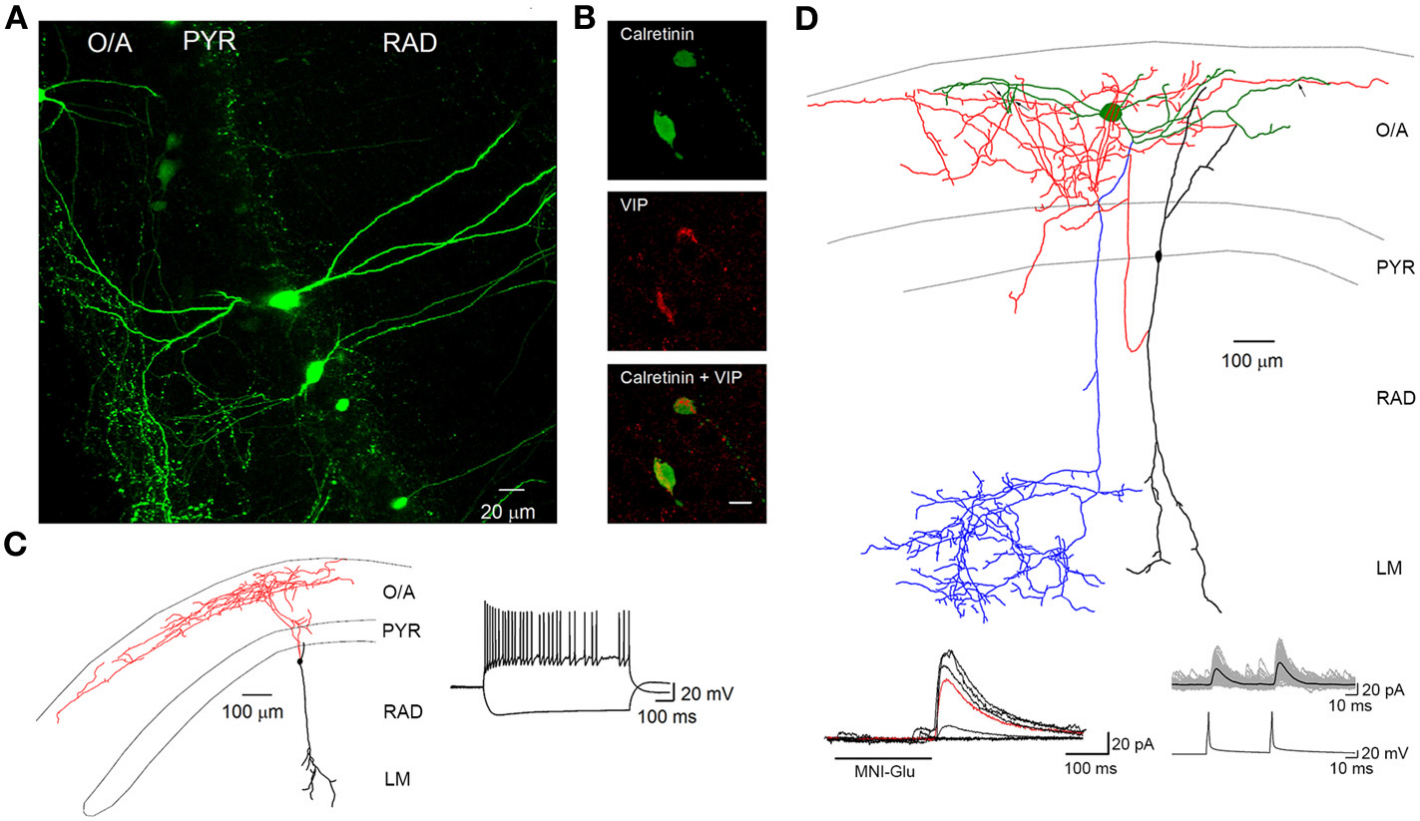
**VIP-positive interneurons at the PYR/RAD border target O–LM interneurons. (A)** Maximal projection of a two-photon z-stack acquired in the CA1 region of the hippocampus of a VIP-eGFP mouse, showing bipolarly oriented VIP-positive cell bodies located at the PYR/RAD border and a dense axonal arborization in the O/A. **(B)** Immunofluorescence images of neurons located in PYR positive for calretinin (top) and VIP (middle) as well as their superimposition (bottom). Scale bar: 20 μ m. **(C)** Reconstruction of a bipolarly oriented VIP-positive cell, showing anatomical features of IS-IIIs (soma and dendrites are shown in black and axon is shown in red) and its irregularly spiking firing pattern typical for these cells. **(D)** Neurolucida reconstruction of a connected pair of interneurons: presynaptic IS-III (soma and dendrites are in black and axon is in red) and postsynaptic O–LM (soma and dendrites are in green, axon is in blue) and examples of unitary IPSCs evoked by two-photon glutamate uncaging (bottom left) and presynaptic spikes during paired recordings (bottom right). Black arrows indicate three putative contact sites onto O–LM dendrites. Modified from Chamberland et al. (2010).

IS interneurons type III (IS-IIIs) are typically located at the PYR and RAD border and has a vertically oriented cell body with dendrites extending into the LM. Their axons form a dense plexus in the O/A (Acsady et al., 1996; Gulyas et al., 1996; Chamberland et al., 2010), where they provide multiple contacts with dendrites of horizontally oriented mGluR1α-expressing interneurons. IS-IIIs coexpress CR and VIP (Gulyas et al., 1996) and may also express enkephalins and the substance P receptor (Freund and Buzsaki, 1996; Blasco-Ibanez et al., 1998). In addition, IS-IIIs are positive for mGluR1α (Ferraguti et al., 2004) and their axonal terminals are decorated with mGluR7 (Somogyi et al., 2003). In contrast to IS-Is, IS-IIIs form synapses mainly onto SOM- and mGluR1α-expressing O–LMs (Baude et al., 1993; Ferraguti et al., 2004; Chamberland et al., 2010), but may also contact other CB-positive interneurons located in the O/A (Figure 1). Our recent findings revealed a functional connection between cells corresponding to IS-IIIs and O–LMs (Chamberland et al., 2010). Using a combination of **two-photon glutamate uncaging-based photostimulation** and patch-clamp recordings in slices obtained from VIP-eGFP mice, we found that the activation of VIP-positive interneurons located at the border between the PYR and the RAD produces small-amplitude and kinetically slow inhibitory postsynaptic currents (IPSCs) in O–LMs. After the uncaging experiments, VIP-positive interneurons connected to O–LMs were patched and filled with biocytin. Further electrophysiological, anatomical, and neurochemical analyses revealed that putative IS-IIIs exhibit a high input resistance and an irregularly spiking firing pattern, establish several synaptic contacts onto dendrites of O–LMs, and coexpress CR (Figure 2) (Chamberland et al., 2010).

In summary, IS interneurons represent a unique population of inhibitory cells, which, via executive control of other interneurons, may provide a higher level of coordination of hippocampal network activity. This is in contrast to neocortical CR-positive interneurons, which preferentially innervate other interneurons, but also contact principal cells. Interestingly, CR-positive interneurons in the neocortex and the hippocampus have a common origin within the caudal ganglionic eminence (Xu et al., 2004; Tricoire et al., 2011); however, as they are integrated in different circuits, these cells may choose different communication partners. The circuit-guiding mechanisms that are responsible for the integration of distinct types of interneurons and their target selectivity remain to be determined.

## Long-range GABAergic projections

In addition to the local circuit inhibitory connections, the activity of hippocampal interneurons can be controlled by extrinsic GABAergic projections: one arising from the medial septum (MS) (Freund and Antal, 1988) and the other from the medial entorhinal cortex (MEC) (Germroth et al., 1989a,b). First, anterograde labeling together with immunohistochemistry showed that septohippocampal GABAergic projections originating from PV-positive interneurons located in the MS innervate the perisomatic region of various types of hippocampal interneurons (Freund and Antal, 1988). As a result, activation of septal GABAergic afferents produced a silencing of interneurons and was associated with disinhibition in pyramidal cells (Toth et al., 1997). Remarkably, two distinct populations of MS interneurons have been identified: fast-firing and burst-firing cells (Jones et al., 1999; Morris et al., 1999; Knapp et al., 2000; Sotty et al., 2003; Simon et al., 2006; Manseau et al., 2008). A subset of these cells express hyperpolarization-activated and cyclic-nucleotide-gated non-selective cation channels and exhibit I_h_ and rebound spiking in response to rhythmic inhibition (Sotty et al., 2003; Borhegyi et al., 2004; Manseau et al., 2008). Furthermore, MS interneurons show a different phase preference during hippocampal theta activity *in vivo* and may target different types of hippocampal interneurons (Dragoi et al., 1999; Borhegyi et al., 2004). In particular, cells that are active at the positive peak of the theta oscillation control dendritic inhibition of CA1 pyramidal cells (e.g., O–LMs) (Borhegyi et al., 2004). Recently, we found that the MS inhibitory input is able to provide large-amplitude sustained perisomatic inhibition to O–LMs during theta-like activity and, therefore, is ideally suited for suppressing O–LM activity at the positive peak of the theta wave (Chamberland et al., 2010).

Second, a bidirectional GABAergic connection between the MEC and the hippocampus has been identified (Germroth et al., 1989a,b; Melzer et al., 2012). In particular, a combination of electrophysiological and optogenetic approaches revealed that a MEC GABAergic projection originating primarily from PV-positive cells can preferentially target interneurons located in the LM (Melzer et al., 2012). This is in contrast to the widespread arborization of septal GABAergic projections in the CA1 region (Freund and Antal, 1988). The preferential innervation of interneurons in the LM supports a specific role for the GABAergic MEC input in controlling the feedforward inhibition that operates in conjunction with the perforant pathway and is necessary for the coordination of activity across different cortical structures.

In summary, hippocampal interneurons receive GABAergic inputs from three main sources: non-specific local inhibitory connections, IS interneurons, and long-range inhibitory projections. Some inhibitory microcircuits, which represent a single class of interconnected interneurons, can be considered as relatively autonomous. For example, dentate gyrus PV-positive BCs are connected in a tight network that is sufficient for generating oscillations in the gamma range (Bartos et al., 2001, 2002). Other inhibitory microcircuits (e.g., composed of CCK-positive BCs or O–LMs) may require a higher level of control via selective activation of IS cells. Finally, the activity of hippocampal inhibitory circuits is tuned via long-range GABAergic projections, providing a means for the tight coordination of activity between connected brain structures.

## Properties of inhibitory synapses onto interneurons

Although the same basic mechanisms of GABA_A_ receptor activation operate in principal cells and interneurons, input- and target-cell specificities of synaptic inhibition have been reported. The inhibitory postsynaptic responses recorded in interneurons can be significantly faster than those recorded in principal cells (Ali et al., 1999; Bartos et al., 2001; Patenaude et al., 2005; Ali and Todorova, 2010). For example, paired recordings showed that IPSCs recorded at dentate gyrus BC–BC synapses were twofold faster than those recorded at BC–granule cell synapses (Bartos et al., 2001). Similarly, evoked IPSCs recorded in the hippocampal RAD interneurons were consistently faster than those in pyramidal cells (Patenaude et al., 2005). Furthermore, paired recordings between SC-ACs and their targets showed that inhibitory postsynaptic potentials recorded in SC-ACs were faster than those in pyramidal cells (Ali and Todorova, 2010). Conversely, spontaneous IPSCs recorded in different subclasses of O/A interneurons decayed more slowly than those in principal cells (Hajos and Mody, 1997). Together, these findings indicate that inhibitory synapses formed onto interneurons may exhibit cell-type-specific properties. What mechanisms can account for such differences in the properties of synaptic inhibition?

First, different location of various inhibitory synapses along the dendritic tree and associated electrotonic attenuation can explain kinetic variations of inhibitory currents in different targets. In fact, even in a given interneuron the rise time and decay of inhibitory currents can vary over 10-fold as a result of dendritic filtering (Hajos and Mody, 1997). In addition, the kinetics of the inhibitory current associated with the opening of the GABA_A_ receptor channel is determined by the subunit composition of the receptor. For example, the presence of the α1 or α6 GABA_A_ receptor subunits is associated with a faster current (Tia et al., 1996; Dunning et al., 1999; Okada et al., 2000; Bosman et al., 2005). In contrast, the presence of the α5 subunit is responsible for the generation of a slower current (Bartos et al., 2004; Ali and Thomson, 2008; Salesse et al., 2011). In the hippocampus, the expression of five α subunits (α1–5) and β1, β3, γ2, and δ subunits has been reported (Sperk et al., 1997). Interestingly, hippocampal interneurons were shown to express a wide spectrum of GABA_A_ receptor subunits, supporting the heterogeneity of the properties of their inhibitory synapses (Gao and Fritschy, 1994; Nusser et al., 1995; Patenaude et al., 2001). Accordingly, immunohistochemical analysis revealed that, in contrast to pyramidal cells, which coexpress the α1, α2, and α5 subunits (the α5 subunit being expressed extrasynaptically), some interneurons express preferentially the α1 GABA_A_ receptor subunit at their synapses, which may account for the faster IPSC kinetics observed in these cells (Gao and Fritschy, 1994; Fritschy and Mohler, 1995; Nusser et al., 1996; Bartos et al., 2001, 2002; Ali and Todorova, 2010). In other subclasses of interneurons (e.g., O–LMs), the α5 GABA_A_ receptor subunit can be incorporated into synapses later during development and is responsible for the age-dependent slowing of IPSCs (Salesse et al., 2011). Furthermore, significant kinetic fluctuations have been observed in interneurons expressing a single type of the GABA_A_ receptor (Nusser et al., 2001). In this case, a significant variability in IPSC decay was associated with the spatiotemporal profile of fluctuations in GABA concentration in the synaptic cleft. In particular, changes in the concentration peak and the speed of GABA clearance have been reported as an important source of synaptic variability (Barberis et al., 2004). For example, the NGFCs provide unusually slow inhibition to their postsynaptic targets due to a specific spatiotemporal profile of GABA near the activated synapses (Karayannis et al., 2010). In addition, NGFCs provide a significantly slower inhibition to CA1 pyramidal cells than to interneurons (Price et al., 2005, 2008) probably as a result of stronger dendritic filtering in principal neurons. Input-specific differences in GABA release have been also reported in different subclasses of hippocampal interneurons (Daw et al., 2009; Chamberland et al., 2010). Anatomical data indicate that septal GABAergic terminals have a larger volume and synapse surface area with a larger number of vesicles than do terminals formed by local GABAergic projections. Furthermore, septal terminals contact their postsynaptic targets via multiple release sites (Eyre et al., 2007). Consistent with these data, we found a sustained reliable transmission during repetitive activity at septohippocampal GABAergic synapses formed onto O–LMs (Chamberland et al., 2010). Finally, cell-type-specific differences in the intracellular chloride concentration can also account for the differences in the amplitude and kinetics of GABAergic currents (Houston et al., 2009). Accordingly, a more depolarized chloride reversal potential was found in CA1 RAD interneurons (−61.3 mV) compared with CA1 pyramidal cells (−66.7 mV) (Patenaude et al., 2005). Moreover, whereas hippocampal circuit maturation is associated with a shift in the chloride reversal potential and, accordingly, the GABA effect in pyramidal neurons, the chloride reversal potential remains unchanged during the maturation of stratum lucidum interneurons and dentate gyrus BCs (Cherubini et al., 1990; Banke and McBain, 2006; Sauer et al., 2012).

Together, these findings highlight the input- and target-specific organization of synaptic inhibition in hippocampal interneurons. In particular, the properties and spatiotemporal profile of transmitter release, the composition of the GABA_A_ receptor, the dendritic filtering and the intracellular chloride concentration shape the dynamics of transmission at different inhibitory synapses. The mechanisms of synaptic inhibition in interneurons and, in particular, the great diversity of the properties of inhibitory synapses within and between different inhibitory circuits remain to be explored.

## Long-term plasticity at inhibitory synapses onto interneurons

The efficacy of GABAergic synapses can be regulated in an activity-dependent manner (Stelzer et al., 1987; Grunze et al., 1996; Nusser et al., 1998; Chevaleyre and Castillo, 2003; Nugent et al., 2007; Xu et al., 2008). Some forms of plasticity found at GABAergic synapses onto pyramidal cells are also present in interneurons. For example, the endocannabinoid-dependent long-term depression (LTD) described at inhibitory synapses onto pyramidal cells also occurs in interneurons (Chevaleyre and Castillo, 2003; Ali, 2007, 2011; Ali and Todorova, 2010). In other cases, the mechanisms of plasticity at GABAergic synapses made onto interneurons can differ significantly from those formed onto principal cells. For example, theta-burst synaptic stimulation induces the long-term potentiation (LTP) of IPSCs in both RAD interneurons and CA1 pyramidal cells, but is regulated via different induction mechanisms. In pyramidal cells, LTP is mediated by the activation of both GABA_B_ receptors and group I/II metabotropic glutamate receptors (Patenaude et al., 2003). In interneurons, the induction of LTP does not require GABA_B_ receptors or group I/II metabotropic glutamate receptors. Furthermore, high-frequency stimulation or postsynaptic depolarization alone produces a short-term depression of IPSCs in principal cells, but not in interneurons (Patenaude et al., 2005). In RAD interneurons, postsynaptic firing at theta frequency is associated with the LTP of IPSCs, which is induced postsynaptically but expressed presynaptically (Evstratova et al., 2011). Although the retrograde messenger required for this LTP induction is unknown, it may involve nitric oxide, which is present in several subclasses of RAD interneurons (Jinno and Kosaka, 2002; Nugent et al., 2007).

These findings highlight the similarities and major differences between specific forms of plasticity at GABAergic synapses formed onto different targets. Such heterogeneity in plasticity mechanisms may result from the cellular and molecular heterogeneity of interneurons and the distinct types of inhibitory inputs that contact these cells. Obviously, understanding these mechanisms will require careful examination of synapses formed by specific inhibitory projections onto the same GABAergic target. To this end, recent data from our laboratory demonstrate the selective strengthening of GABAergic synapses formed by different inputs onto O–LM interneurons (Chamberland et al., 2010; Salesse et al., 2011). Whole-cell and perforated patch-clamp recordings from O–LMs identified in slices from young mice (15 < P < 25) revealed that a 10 Hz stimulation induced postsynaptic LTP of IPSCs at septal synapses, but not at those formed by local GABAergic projections. Interestingly, in slices obtained from mature animals (26 < P < 40), GABAergic synapses formed onto O–LMs by local projections exhibited LTP. The expression of the latter form of LTP correlated well with the synaptic incorporation of the α5-GABA_A_ receptor subunit and LTP was blocked by an α5-GABA_A_ receptor subunit inverse agonist, highlighting the primary role of this subunit in LTP at local synapses.

## Conclusion and future directions

There is growing evidence that the activity of hippocampal inhibitory circuits is tightly regulated by multiple inhibitory mechanisms. First, distinct classes of interneurons are reciprocally interconnected and form closely interacting networks. As proposed by Buzsaki (1997), such networks of inhibitory interneurons impose coordinated oscillatory “contexts” for the “content” carried by the networks of principal cells. Therefore, interneuron interconnectivity has been considered to be a fundamental mechanism in maintaining network oscillations at different frequencies: from relatively slow theta to gamma and higher-frequency ripple oscillations (Bragin et al., 1995; Cobb et al., 1995; Whittington et al., 1995; Traub et al., 1996; Wang and Buzsaki, 1996; Bartos et al., 2001, 2002; Traub et al., 2001). The properties of connections between interneurons, in particular synaptic strength and kinetics, the presence of electrical coupling and spacing between connected interneurons appear to play a major role in the frequency and coherence of network oscillations (Whittington et al., 1995; Traub et al., 1996; Bartos et al., 2002). In addition, the presence of autaptic connections in some interneurons (e.g., PV-positive BCs) increases the spike fidelity in these cells to ensure their temporally precise firing (Bacci and Huguenard, 2006). As a result, fast inhibitory circuits of tightly coupled PV-positive BCs promote synchronized gamma oscillations (Bartos et al., 2001, 2002), whereas slower inhibitory circuits that link O–LMs and LM interneurons have been associated with the generation of theta-frequency outputs (Maccaferri and McBain, 1996; Traub et al., 1998; Chapman and Lacaille, 1999). It is to be noted that depending on the expression of the K^+^/Cl^−^ cotransporter KCC2, the effect of GABA can be depolarizing (Rivera et al., 1999). However, the presence of shunting and depolarizing GABA currents is likely to improve the overall coherence of the interneuronal network by accelerating weakly activated interneurons and decelerating the strongly activated ones (Vida et al., 2006). This will support the generation of fast coherent oscillations even in the absence of strong tonic drive to the interneuronal network (Mann and Paulsen, 2006). Therefore, specific inhibitory mechanisms connecting interneuron nets are built to support their oscillatory behavior. Furthermore, the hippocampus is populated by a large variety of classes of interneurons, with synaptic connections formed within a class as well as between classes. It will now be crucial to examine how these different levels of inhibitory communication are engaged in the intact animal during behavior. Addressing this question will require a set of innovative techniques that combine electrophysiological recordings and labeling of interneurons in freely moving animals. Recent data show that specific classes of hippocampal interneurons can be recorded and labeled in freely moving rats (Lapray et al., 2012), suggesting that the contribution of the various interconnected inhibitory circuits to network activity during behavior will soon be discovered.

Why does a dedicated population of interneurons specialized in the exclusive innervation of other GABAergic cells exist? First, it appears that IS interneurons are highly selective in choosing their targets, suggesting that not all classes of hippocampal interneurons are controlled by IS cells. Interestingly, both IS-Is and IS-IIs prefer to contact CCK-positive BCs, whereas IS-IIIs prefer O–LMs. The reasons underlying such preferences remain to be determined. It is important to note, however, that IS-IIIs are strategically positioned to detect the changes in network activity that are transmitted by the perforant path and, therefore, to adjust the strength of inhibition exerted by the O–LMs on perforant path integration. As O–LMs are not contacted by the perforant path, such an indirect mechanism of regulation of their activity seems functionally plausible. Unfortunately, very little is currently known about the physiological organization of IS interneurons. Thus, even though IS-IIIs exhibit extensively branching dendrites in the LM and are likely to be recruited via activation of the perforant path, this hypothesis has not been tested directly. Therefore, from the functional point of view, IS interneurons may be positioned to control selective inhibitory circuits. Second, IS cells may sense and communicate specific changes in network activity and the environment depending on the brain state. For example, the coexpression of enkephalins (Blasco-Ibanez et al., 1998), 5-HT-3 (Jakab and Goldman-Rakic, 1996; Morales et al., 1996), D1/D2 (Gangarossa et al., 2012), and substance P receptors (Freund and Buzsaki, 1996) in IS interneurons indicates that these cells may detect specific intrinsic and extrinsic influences to control the balance of hippocampal inhibition.

Finally, given that the strength and time course of inhibitory transmission serve to control the recruitment and synchronization of interneurons during network activity, a better understanding of the properties of synaptic transmission between individual inhibitory circuit elements is required. The efficiency of the communication between inhibitory circuits given a relatively sparse connection pattern, the manner via which it may change during different forms of activity, and the manner via which the balance between excitatory and inhibitory inputs is achieved to provide coordinated interneuron activity remain largely unknown. The view that coordinated inhibition is required for the proper routing of excitatory trajectories and for the spatiotemporal organization of hippocampal cell assemblies should inspire further research on the targeting of interconnected inhibitory circuits.

### Conflict of interest statement

The authors declare that the research was conducted in the absence of any commercial or financial relationships that could be construed as a potential conflict of interest.

## Key Concept

GABAergic inhibitory neurons: Non-principal neuron which releases γ-aminobutyric acid (GABA) to inhibit its targets. In the hippocampus, these cells represent both local and projection neurons. At least 21 different types of GABAergic inhibitory cells populate the CA1 area of the hippocampus.
Paired recordings: Electrophysiological recordings from two neurons simultaneously aimed to identify synaptically connected partners. If neurons are connected, generating an action potential (AP) in one cell will evoke a postsynaptic response in the other one. Combination of paired recordings with biocytin labeling allows anatomical identification of synaptically connected neurons.
Interneuron-specific (IS) interneurons: GABAergic interneuron that innervates selectively other GABAergic cells. In the hippocampus, three distinct types of IS interneurons have been identified based on the combination of anatomical and molecular features. IS interneurons can express the calcium-binding protein calretinin, the vasoactive intestinal peptide, or a combination of both.
Two-photon glutamate uncaging-based photostimulation: Optophysiological mapping of synaptically connected neurons with single-cell resolution. Highly-localized excitation of putative presynaptic neurons is achieved by two-photon uncaging of caged glutamate. When combined with electrophysiological recording, this method allows a fast examination of multiple connections to a given neuron.
